# Pathogenesis of influenza and SARS-CoV-2 co-infection at the extremes of age: decipher the ominous tales of immune vulnerability

**DOI:** 10.1007/s44307-025-00057-9

**Published:** 2025-01-21

**Authors:** Kai-lin Mai, Wei-qi Pan, Zheng-shi Lin, Yang Wang, Zi-feng Yang

**Affiliations:** 1 Henan University College of Medicine, Kaifeng, 475004 China; 2 School of Life Sciences, Henan University, Kaifeng, 475004 China; 3 National Clinical Research Center for Respiratory Disease, State Key Laboratory of Respiratory Disease, Guangzhou Institute of Respiratory Health, the First Affiliated Hospital of Guangzhou Medical University, Guangzhou, 510120 China; 4 Guangzhou National Laboratory, Guangzhou, 510005 China

**Keywords:** Influenza, SARS-CoV-2, Co-infection, Immature immunity, Immunosenescence

## Abstract

The co-circulation of influenza and SARS-CoV-2 has led to co-infection events, primarily affecting children and older adults, who are at higher risk for severe disease. Although co-infection prevalence is relatively low, it is associated with worse outcomes compared to mono-infections. Previous studies have shown that the outcomes of co-infection depend on multiple factors, including viral interference, virus-host interaction and host response. Children and the elderly exhibit distinct patterns of antiviral response, which involve airway epithelium, mucociliary clearance, innate and adaptive immune cells, and inflammatory mediators. This review explores the pathogeneses of SARS-CoV-2 and influenza co-infection, focusing on the antiviral responses in children and the elderly. By comparing immature immunity in children and immune senescence in older adults, we aim to provide insights for the clinical management of severe co-infection cases.

## Introduction

The global pandemic of coronavirus disease of 2019 (COVID-19) caused by the severe acute respiratory syndrome coronavirus 2 (SARS-CoV-2) has posed an unprecedented challenge to the public health since late 2019 (Zhu et al. 2020). Although the World Health Organization declared it is no longer a public health emergency on May 11, 2023 (Harris 2023), the virus continues to circulate with the constant emergence of new SARS-CoV-2 variants (Callaway 2023). Meanwhile, the return of seasonal influenza activity to pre-pandemic levels, following its brief decline during the pandemic, has raised renewed awareness (Chow et al. 2023; Wang and Wang 2023). As a result, the co-circulation of SARS-CoV-2 and the influenza virus can inevitably lead to co-infections in humans, causing more complex diseases and potentially resulting in severe outcomes (Zheng et al. 2020; Yang et al. 2022; Krumbein et al. 2023).

The prevalence of co-infection of SARS-CoV-2 and influenza virus in humans may vary geographically and be affected by non-pharmaceutical interventions to control SARS-CoV-2 transmission across different places and time periods (Dadashi et al. 2021; Dao et al. 2021; Costa et al. 2023; Krumbein et al. 2023; Yan et al. 2023). When COVID-19 first appeared, it coincided with the high seasonal influenza activity in China; during this period, over 40% of confirmed COVID-19 cases tested positive for serum IgM against influenza A virus and 7.5% positive against influenza B (Yue et al. 2020; Cheng et al. 2021). Around the same time in Iran, 22% of deceased COVID-19 patients tested positive for influenza infection (Hashemi et al. 2021). The historically low levels of within-season influenza activity that were seen in 2020 and 2021 as a result of the widespread non-pharmaceutical interventions subsequently resulted in a lower co-infection rate, while SARS-CoV-2 infection was still predominant (Maltezou et al. 2023).

A recent meta-analysis found that influenza A virus was the most often co-infected virus with SARS-CoV-2, with a pooled prevalence of 2.45% (95% CI: 1.67–3.58%), ranging from 0% to 57.33% (Yan et al. 2023). Notably, youngsters and the elderly have shown higher co-infection rates than adults (Maltezou et al. 2023; Yan et al. 2023). Additionally, co-infections with SARS-CoV-2 and other viruses were more prevalent in pediatric patients compared to adults (Krumbein et al. 2023). Clinically, co-infection of SARS-CoV-2 with influenza virus may exacerbate organ damage, particularly in the lung. Patients with co-infections often experience the most severe complications, such as respiratory distress syndrome and multi-organ dysfunction (Maltezou et al. 2023), and had a higher incidence of acute kidney and cardiac injuries compared to SARS-CoV-2 mono-infection (Ma et al. 2020; Zheng et al. 2021). Specifically, COVID-19 patients with influenza co-infection had an increased risk of requiring mechanical ventilation and admission of intensive care unit (ICU) compared to those without co-infection (Garg et al. 2022; Yan et al. 2023), and co-infected pediatric patients also faced a higher risk of requiring mechanical ventilation compared to SARS-CoV-2 mono-infection (Eşki et al. 2021; Adams et al. 2022). An animal study found that golden Syrian hamsters co-infected with SARS-CoV-2 and A(H1N1)pdm09, either simultaneously or sequentially, caused more severe disease than those infected with either virus alone (Zhang et al. 2021).

The higher incidence and a poor outcome of co-infection with influenza virus and SARS-CoV-2 in children and the elderly suggest the possibility of age-related pathogeneses, with distinct immune responses at the extremes of age potentially playing a key role (Grudzinska et al. 2020). This review will explore why children and the elderly are more vulnerable to SARS-CoV-2 and influenza co-infection from the perspectives of underdeveloped immunity in youth and immunological senescence in the elderly.

## Factors affecting severity of influenza and SARS-CoV-2 co-infection

To understand the pathogenesis of viral co-infection, it is important to first consider how these viruses interact with each other and the host. From the viral perspective, the outcomes of viral co-infection are typically influenced by factors such as virus dose, the time interval between co-infecting viruses, cell type, and viral replication rate (Kumar et al. 2018). In the case of influenza and SARS-CoV-2 co-infection, the interference between the two viruses, along with the broader virus-host interactions, can significantly affect disease severity.

### Interference between influenza and SARS-CoV-2

The viral interference phenomenon occurs when one virus competitively suppresses the replication, virulence, and disease severity of the co-infecting virus, leading to reduced cell death and replication of the latter (Piret and Boivin 2022). In studies with varying cell types, virus doses, sequences, and time intervals of co-infection, most findings show that SARS-CoV-2 growth is more prone to interference by influenza, with limited reciprocal interference observed (Achdout et al. 2021; Zhang et al. 2021; Oishi et al. 2022). Regardless of whether the SARS-CoV-2 dose was higher or lower than that of influenza, its viral replication and RNA load were both inhibited. This is consistent with the lower replication rates of SARS-CoV-2 in primary human alveolar and tracheobronchial tissues compared to influenza virus (Zarkoob et al. 2022). On the other hand, SARS-CoV-2 delayed the clearance of influenza virus in murine models, which may contribute to the increased severity of co-infection (Pinky et al. 2023).

### Impact of virus-host interplay on co-infection

Viral interference is commonly mediated by interferons (IFNs), which had elaborate interplay with both SARS-CoV-2 and influenza viruses. On one hand, SARS-CoV-2 replication is sensitive to IFN treatment, so IFN antiviral responses induced by influenza can significantly inhibit SARS-CoV-2 growth. On the other hand, the IFN antagonistic effect of SARS-CoV-2 prevents it from eliciting sufficient IFN to affect the infectivity of influenza (Essaidi-Laziosi et al. 2022). The absence of interference in the blockade of IFN or in IFN-incompetent cells highlights the crucial role of the IFN response in SARS-CoV-2 and influenza co-infection (Fage et al. 2022; Oishi et al. 2022). Moreover, the parallel exacerbation of the inflammatory response and lung injury during co-infection indicates that disease severity is determined by the host response (Zhang et al. 2021; Kim et al. 2022; Svyatchenko et al. 2023).

SARS-CoV-2 and influenza have adopted multifaceted strategies to evade IFN induction and relevant signaling pathways (Piret and Boivin 2022). With such a broad range of evasion approaches, the effect of viral interference becomes unpredictable. Unlike in experimental conditions, individuals in clinical settings are rarely immunologically quiescent, and their complex immunological histories may influence viral clearance efficiency, contributing to population heterogeneity (Baker et al. 2024).

Additionally, co-infection can affect the expression of viral receptors. Specifically, SARS-CoV-2 and influenza viruses could mutually increase each other's entry receptor expression on host cells (Kim et al. 2023). In the human lung organoids, infection of SARS-CoV-2 delta variants upregulated α−2–3-linked sialic acid, while influenza infection increased the expression of angiotensin-converting enzyme 2 (ACE2) and transmembrane serine protease 2 (TMPRSS2). Co-infection with SARS-CoV-2 and influenza virus in the human lung organoids resulted in reciprocal enhancement of viral replication.

## Antiviral response at the extremes of age

**Fig. 1:**
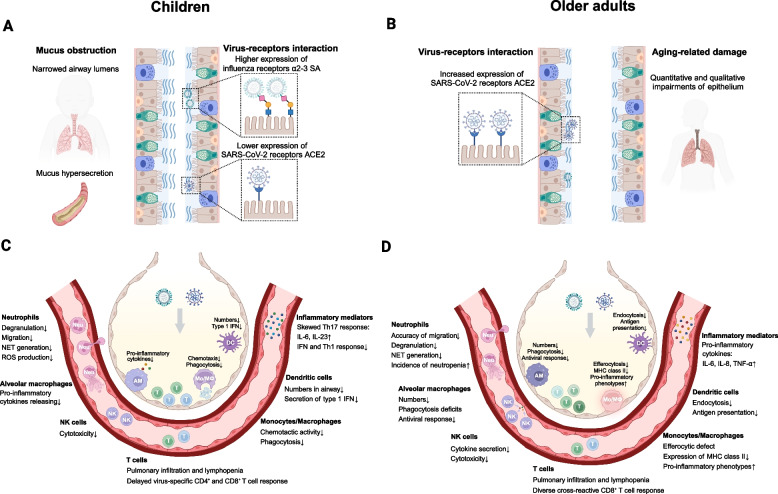
Characteristics of host antiviral responses in upper respiratory tract (A and B), lower respiratory tract and peripheral blood (C and D) in children and older adults, respectively. **A** and **B**, the upper respiratory tracts of children are characterized by narrowed airway lumens and mucus hypersecretion; while older adults have aging-related epithelial damages. Dotted black boxes illustrate the distinct receptor patterns of SARS-CoV-2 and influenza in the upper airway of children and older adults. **C** and **D**, alterations in immune cell functions and cytokine responses that may contribute to severe immunopathology in children and older adults during pulmonary viral infections. Abbreviations: SA, sialic acid; ACE2, angiotensin-converting enzyme II; Neu, neutrophils; NET, neutrophil extracellular traps; ROS, reactive oxygen species; AM, alveolar macrophages; NK, natural killer cells; Mo/MΦ, monocytes/macrophages; DC, dendritic cells

Children and the elderly are often considered vulnerable populations at greater risk of viral infections and co-infections, but their effects on disease severity remains debated (Scotta et al. 2016). During the COVID-19 pandemic, children infected with SARS-CoV-2 generally experienced a milder disease course and better outcomes, consistent with previous observations of SARS-CoV infection (Stockman et al. 2007). In contrast, older adults are more likely to suffer from age-related diseases, leading to a higher prevalence of comorbidities and increased disease severity (Maltezou et al. 2023). While inborn errors of immunity provide alternative explanations, genetic susceptibility accounts for only a small proportion of critical influenza or COVID-19 cases (Casanova and Abel 2022). This topic will not be further addressed here, as it has been reviewed elsewhere (Darbeheshti et al. 2021). To compare the host responses to influenza and SARS-CoV-2 infections in children and older adults, we summarize the roles of the airway epithelium, mucociliary clearance, innate and adaptive immune cells, and inflammatory mediators (Fig. 1).

### Airway epithelium and mucociliary clearance

#### Composition of airway epithelium

As the first line of defense, airway epithelium and the mucociliary escalator provide barrier-protective effects against inhaled viral particles. The intact epithelium, composed of epithelial cells and intercellular junctions, overlaid by the mucus layer in contact with the air, serves as a physical barrier. Normally, most inhaled particles are transported from the bronchioles to the upper respiratory tract along with mucus, through the ciliary motion of ciliated epithelial cells. Airway mucus is a viscous fluid secreted by goblet cells in the epithelium and submucosal glands, containing various antimicrobial and antioxidant proteins (Vareille et al. 2011). The primary component of airway mucus is mucins, a family of heavily O-glycosylated and high-molecular weight glycoproteins, which contains sialic acids that can bind hemagglutinin (HA) protein of influenza virus and potentially interact with spike glycoprotein (S-protein) of SARS-CoV-2 (Couceiro et al. 1993; Unione et al. 2022). The presence of human mucus inhibiting both swine and human influenza A virus infections in MDCK cell cultures suggests that human respiratory mucus may prevent viral contact with the epithelium by trapping the viruses (Zanin et al. 2015). To overcome this, human-adapted influenza viruses possess a long-stalk neuraminidase (NA) that cleaves sialic acids, allowing the virus to escape mucus (Zanin et al. 2016; Long et al. 2019). Located closest to the airway epithelium, a thin layer of surfactant is secreted by alveolar type II cells and Club cells, primarily coating the alveolar epithelium. The hydrophobic surfactant proteins (SP-B and SP-C) interact with lipids to reduce surface tension and prevent lung collapse (Isasi-Campillo et al. 2023). The hydrophilic surfactant proteins (SP-A and SP-D) contain a carbohydrate recognition domain that binds to microbial carbohydrate epitopes, exerting antiviral effects by binding to the HA of influenza virus and the S-protein of SARS-CoV-2. However, surfactant proteins in older adults have been reported to exhibit reduced pathogen-binding capacity (Benne et al. 1995; Hartshorn et al. 2008; Moliva et al. 2019; Jacob et al. 2024).

#### Virus-receptor interaction

When viral particles pass through the mucus barrier and reach the epithelial cells, surface glycoproteins of influenza and SARS-CoV-2 bind to specific receptors for attachment and entry. The HA of human influenza virus recognizes α2–6 sialic acid (SA) receptors, which are mainly found in the upper respiratory tract. In contrast, avian influenza viruses, which are more pathogenic in humans, prefer α2–3 SA receptors in the lower respiratory tract (Long et al. 2019). Although there is evidence of interaction between SA and S-protein, the primary receptor for SARS-CoV-2 is ACE2. Additionally, TMPRSS2 is also required for SARS-CoV-2 entry (Hoffmann et al. 2020; Nguyen et al. 2022). Age-dependent differences in receptor distribution for both viruses may account for variations in susceptibility and severity. In comparison to adults, children have higher expression of α2–3 SA in the respiratory tract, which could partly explain their increased susceptibility to avian influenza H5N1 during previous outbreaks (Nicholls et al. 2007; Uyeki 2008). In contrast, *ACE2* gene expression in the nasal epithelium and ACE2 protein expression in both alveolar epithelial cells and lung tissues were lower in children compared to adults (Bunyavanich et al. 2020; Silva et al. 2023). Additionally, older males exhibited higher levels of ACE2 in several organs, as observed in both mice and humans (Viveiros et al. 2022). These variations in host receptor distributions correspond with the observed increase in COVID-19 hospitalization and mortality rates with age (Romero Starke et al. 2021).

#### Epithelial injury and repair

Once the glycoproteins of influenza and SARS-CoV-2 recognize and attach to cell surfaces, various host immune defenses are triggered, including protection against viral infection and the eradication of established infection. Killing infected cells helps protect uninfected cells, while the severity of lung injury is determined by the extent of epithelial cell death (Atkin-Smith et al. 2018; Yuan et al. 2023). Comparisons of viral replication levels in nasal epithelial cell cultures from young and older hosts revealed that, after influenza infection, viral replication was similar in both groups (Chason et al. 2018). Therefore, it is hypothesized that the baseline characteristics of the epithelium influence the differing patterns of damage and repair between children and the elderly. Upon birth, epithelium differentiation and blood-gas barrier have completely developed (Stocks et al. 2013). Alveolarization continues through childhood and adolescence, suggesting that the process of lung development could support epithelial repair after viral infection (Narayanan et al. 2012). In older adults, both quantitative and qualitative impairments in the cellular composition of the airway epithelium occur with age. With diminished self-renewal, reduced mucociliary clearance, and a decreased gas exchange area, the aging lung is more susceptible to injury, with slower viral clearance and tissue repair (Schneider et al. 2021).

#### Mucus obstruction

Severity of post-infection respiratory symptoms is associated with airway mucus. Respiratory viruses can stimulate mucin production, which inhibit replication by effectively binding viral particles (Ehre et al. 2012; Zanin et al. 2016). Nevertheless, excessive mucus secretion is detrimental to both children and the elderly, especially since children have more mucous glands than adults (Di Cicco et al. 2021). In an autopsy of an infant co-infected with SARS-CoV-2 and influenza, a viscous mucus plug was observed from the bronchioles to the alveoli (Jeican et al. 2023). Under normal homeostatic conditions, the balance between mucus production and the mucociliary clearance system allows the cilia on the airway epithelium to efficiently move mucus and trap substances for swallowing. However, with increased mucus production, coughing becomes necessary to propel the mucus towards the pharynx (Munkholm and Mortensen 2014). Cough efficacy, which involves both the nervous system and respiratory muscle activation, is age-dependent. In the early months of life, infants have an immature cough reflex, whereas the elderly experience weakened cough strength due to respiratory muscle atrophy (Munkholm and Mortensen 2014; Nagano et al. 2021). Additionally, both children and older adults are vulnerable to overproduction of mucus. Older adults have increased alveolar surface tension and decreased lung compliance due to reduced lipid content in the airways, while children are at higher risk due to narrower airway lumens (Di Cicco et al. 2021; Esfehani et al. 2021). As a result, both children and the elderly are prone to airway obstruction and reduced gas exchange efficiency.

### Innate immune cells

In brief, innate immune cells are crucial for inhibiting viral replication, clearing infected cells and boosting the viral-specific adaptive immune response (Sievers et al. 2024). Uncontrolled viral growth can aberrantly recruit and activate innate immune cells, triggering excessive inflammation and leading to immunopathology (Mazzoni et al. 2021). Both children and the elderly exhibit hypofunctional innate immune cells compared to healthy adults, characterized by distinct patterns of dysregulated immune activation and inflammatory microenvironments (Kollmann et al. 2012; Grudzinska et al. 2020).

#### Alveolar macrophages

Alveolar macrophages (AMs) in the airspaces continuously phagocytose inhaled exogenous substances to maintain lung homeostasis, serving as first-line defenders against viral invasion of the alveoli (Wang et al. 2022). AMs develop postnatally and are present in neonates within 48 h of birth, maintaining self-renewal throughout life (Evren et al. 2020; Schneider et al. 2021). In infants, immature AMs are less effective at releasing pro-inflammatory cytokines like TNF-α and IL-1, which are crucial for viral clearance (Grigg et al. 1999; Diamond and Kanneganti 2022). In older adults, both the frequency and function of AMs decline, impairing phagocytosis, scavenging, and antiviral responses, including a reduced ability to respond to IFN-γ signaling (Schneider et al. 2021).

#### Neutrophils

As primary effector cells, circulating neutrophils migrate to infected sites, where they clear viruses and infected cells through phagocytosis, oxidative burst, and neutrophil extracellular trap (NET) formation (Johansson and Kirsebom 2021). However, both children and the elderly exhibit reduced ability to migrate, degranulate, and generate NETs (Grudzinska et al. 2020). Notably, as granulocytic precursors show a diminished response to G-CSF with age, older adults experience a higher incidence of neutropenia during severe infections (Chatta et al. 1993). Moreover, the decreased phagocytic ability and impaired migratory accuracy of neutrophils can result in the paradox of excessive inflammation and inadequate immune defense (Drew et al. 2018).

#### Monocytes/Macrophages

Monocytes, a heterogeneous group of innate effector cells, are activated and recruited by virus-induced inflammatory mediators to infiltrate infected sites. They differentiate into macrophage phenotypes, playing key roles in modulating the inflammatory microenvironment, performing efferocytosis, presenting antigens, and promoting tissue repair (Knoll et al. 2021). Monocytes in infants exhibit impaired chemotaxis and phagocytosis (Georgountzou and Papadopoulos 2017). However, the surface expression of T-cell activation mediators, such as CD80 and HLA-DR, reaches adult levels within the first year, indicating a rapid development of antigen presentation capacity early in life (Nguyen et al. 2010). In older adults, monocyte/macrophages tend to adopt pro-inflammatory phenotypes more readily (Thevaranjan et al. 2017). Additionally, the age-related decline in T-cell immunoglobulin mucin receptor-4 (TIM-4) expression may impair the clearance of apoptotic cells induced by infection (De Maeyer et al. 2020). The efferocytic defect impairs the clearance of apoptotic cells, leading to sustained inflammation, which may contribute to inflammaging (De Maeyer and Chambers 2021). Furthermore, reduced MHC class II expression on monocytes in older adults results in inefficient antigen presentation, compromising the subsequent virus-specific T cell response (Villanueva et al. 1990).

#### Dendritic cells and natural killer cells

As the most potent antigen-presenting cells, dendritic cells (DCs) play a central role in orchestrating the antiviral response (De Leeuw and Hammad 2024). Although infants have lower densities of respiratory DCs compared to adults (Tschernig et al. 2006), the total number of HLA-DR^+^ cells, with approximately 50% being various DC subsets, increases during the first two years of life (Heier et al. 2008; Georgountzou and Papadopoulos 2017). The ability of plasmacytoid dendritic cells to secrete type 1 IFN is reduced at birth, reaching adult-level function a few weeks postnatally (Nguyen et al. 2010). In older adults, while the number and phenotype of DCs remain largely unchanged, their capacity for endocytosis, antigen presentation, and T cell priming declines (Agrawal and Gupta 2011). Natural killer (NK) cells, which recognize and kill virus-infected cells through the release of cytotoxic granules, exhibit inherent dysfunction in cytotoxicity in newborns (Guilmot et al. 2011). In older adults, an age-related accumulation of abnormal NK cells has been observed, which are impaired in both cytokine secretion and target cell cytotoxicity (Brauning et al. 2022).

### Adaptive immune cells

Adaptive immunity provides pathogen-specific, memory-based defense, determining the outcome of viral infections—either resolution or immunopathology (Nguyen et al. 2024). The rapid innate response, activated within hours post-infection, provides a critical window for the development of virus-specific responses, which can take days or weeks (Mettelman et al. 2022). Lung-resident DCs capture viral antigens, migrate to draining lymph nodes, and interact with B and T cells for antigen presentation. Effector and memory T cells and B cells then mature, expand, and migrate to infection sites, where cytotoxic T cells eliminate infected cells and plasma B cells produce virus-specific antibodies (Prigge et al. 2020; Mettelman et al. 2022). A timely T cell response is crucial for recovery from influenza and COVID-19 (Yunis et al. 2023). However, widespread innate cell dysfunction in both children and the elderly impairs adaptive antiviral responses.

#### Pulmonary T-cell infiltration in severe co-infection

Severe influenza and COVID-19 patients exhibit delayed and diminished virus-specific CD4^+^ and CD8^+^ T cell responses compared to mild cases (Nguyen et al. 2021; Yu et al. 2023). Severe COVID-19 patients also show a delayed innate response, with persistently low levels of T and B cells in peripheral blood. This severe lymphopenia is linked to significant lymphocyte aggregation at the infection site (Bolouri et al. 2021). Autopsy of a 7-month-old infant who died from respiratory and multi-organ failure due to SARS-CoV-2 and influenza co-infection revealed persistent lung inflammation, primarily driven by peribronchial CD20^+^ B cell and perivascular CD3^+^ T cell infiltration (Jeican et al. 2023). Mouse models further confirmed that co-infection resulted in higher levels of CD4^+^ and CD8^+^ T cells in the lungs compared to mono-infection (Kim et al. 2022).

#### Virus-specific T cell response

Age-related changes in adaptive immunity impact antigen specificity and memory development, which depend on postnatal exposure to various pathogens (Kollmann et al. 2012). In early life, T cells have a limited ability to generate memory, leaving infants vulnerable to potentially life-threatening infections (Pieren et al. 2022). Upon antigen stimulation, naïve CD4^+^ T cells in adults primarily differentiate into Th1 cells, which secrete IFN-γ and TNF-α to mediate antiviral responses. In contrast, neonates exhibit a skewed Th2 response with reduced Th1 activity, compromising antiviral efficacy (Moran et al. 1996; Aleebrahim-Dehkordi et al. 2022). Children, compared to adults, exhibit a delayed but intact influenza-specific CD4^+^ T cell response, leading to suboptimal activation of CD8^+^ cytotoxic T lymphocytes (CTLs) and prolonged inflammation (Prigge et al. 2020). In older adults, both senescent antigen-presenting cells and T cells impair the antiviral response (Bartleson et al. 2021). However, their broader T cell repertoire, shaped by multiple antigen exposures, allows rapid CTL memory recall (Quiñones-Parra et al. 2016). Cross-reactive CD8^+^ T cells from prior influenza exposure may offer protection, but weak or non-cross-reactive T cell responses can contribute to severe immunopathology (McAuley et al. 2015). Studies have shown that weak cross-reactive CD8 + T cells can worsen disease in co-infections like influenza and lymphocytic choriomeningitis virus (Wlodarczyk et al. 2013). With limited shared epitopes between SARS-CoV-2 and influenza, CD8^+^ T cell cross-protection is minimal (Lee et al. 2020). Consequently, in older adults, co-infection with SARS-CoV-2 and influenza may lead to broad but low-affinity memory T cells, resulting in inefficient viral clearance and potential immunopathology (Welsh and Fujinami 2007), a phenomenon less likely in children due to their undeveloped immunologic memory.

#### Antibody response

Antibodies, primarily produced by B cells, are integral to adaptive immunity and are crucial in neutralizing viruses. Antiviral antibody responses differ between children and the elderly due to age-related immunological features. In children, antibody production in response to infections like influenza and SARS-CoV-2 is generally robust. Compared to adults, the neutralizing antibody titers are elevated in children (Karron et al. 2022), though this response tends to be more narrowly specific. Although children's antibodies may provide prolonged protection against particular strains of the virus (Bonfante et al. 2021), their response is frequently limited in breadth, rendering it potentially less effective against variants or different subtypes (Meade et al. 2020). In contrast, the elderly experience a decline in both the quantity and quality of antibody production due to immunosenescence. This leads to less affinity and fewer potent neutralizing antibodies, hindering their capacity to establish a robust response against co-infections (Frasca and Blomberg 2020). Thus, the age-associated deterioration in antibody response may exacerbate the severity of viral co-infections in older adults.

### Inflammatory mediators

#### Pattern recognition receptors on immune cells

During viral entry and replication, pattern recognition receptors (PRRs) on innate immune cells detect viral components, triggering cytokine production and the clearance of infected cells. Research on the age-related differences in PRRs distribution is limited and most of them focused on Toll-like receptors (TLRs). TLRs are one of the major classes of PRRs that recognize influenza and SARS-CoV-2, play a key role in the inflammatory response and innate-adaptive immune crosstalk (Chen et al. 2018; Mifsud et al. 2021; Diamond and Kanneganti 2022). The expression of TLRs in healthy infants reaches adult levels by around five years of age. In contrast, the elderly exhibit reduced levels of most TLRs, except for TLR5 (Kollmann et al. 2012). In neonates, diminished TLR-mediated reactive oxygen species (ROS) production impairs viral clearance by neutrophils (Maddux and Douglas 2015; Johansson and Kirsebom 2021).

#### Cytokine profiles in infants and children

Cytokine profiles, as a large family of intercellular signaling molecules, are key indicators of the intensity of host defense responses. Upon TLR stimulation, whole blood and mononuclear cells from healthy term infants primarily produce IL-6 and IL-23, which promote the differentiation of Th17 cells (Kollmann et al. 2009; Corbett et al. 2010). Compared to healthy adults, children exhibit lower levels of IFN and Th1-supporting cytokines, leading to an impaired virus-specific adaptive immune response (Kollmann et al. 2012; Coates et al. 2015). Newborns' cord blood has been shown to have reduced IFN-α/β production in stimulated dendritic cells, due to impaired nuclear translocation and compromised activation of the interferon regulatory factor family (Aksoy et al. 2007; Danis et al. 2008). Additionally, lower reactivity of CD4^+^ and CD8^+^ T cells to viral proteins contributes to reduced IFN-γ levels in children, as IFN-γ is a key antiviral signaling molecule primarily produced by T cells and NK cells (You et al. 2008; Shannon et al. 2019).

#### Inflammaging in older adults

Pro-inflammatory cytokines increase with age, a phenomenon known as inflammaging (Xia et al. 2016). The decline in adaptive immunity, coupled with chronically activated innate immunity, may explain this non-resolving inflammation (Salminen et al. 2008). Triggered by oxidative stress, dysbiosis, and inflammatory cell death, circulating cytokine levels are elevated in the elderly (Li et al. 2023). Higher levels of pro-inflammatory cytokines such as IL-6, IL-8, and TNF-α in aged COVID-19 patients are linked to greater severity and higher mortality risk (Del Valle et al. 2020). Conversely, high IL-10 production is associated with healthy aging and longevity (Lio et al. 2002). Paradoxically, IL-10-producing T follicular helper cells accumulate with age, limiting influenza vaccine efficacy, a process that can be reversed by transient IL-10 blockade (Almanan et al. 2020). The co-elevation of both pro-inflammatory and anti-inflammatory cytokines reflects a complex imbalance in cytokine production in older adults, which impairs the tissue repair process after viral infections.

## Discussion

In summary, age-related differences in immune responses markedly influence the severity and outcome of influenza and SARS-CoV-2 infections. Children encounter delayed immune activation and inefficient viral clearance due to underdeveloped immunity, whereas older adults have immunosenescence, characterized by chronic inflammation and impaired antiviral responses. Both age groups show imbalanced cytokine profile, impeding efficient resolution. These age-dependent immunological traits underscore the necessity for customized therapeutic strategies to improve viral infection outcomes in both populations.

The clinical and immunological impacts of influenza and SARS-CoV-2 co-infections remain controversial in real-world studies. Unlike mono-infection, the severity of viral co-infections is influenced by interactions between the viruses, such as the co-infection interval and differences in replication rates (Kumar et al. 2018). These factors can shape the host immune response. However, viral influences are best controlled in experimental settings. While animal studies consistently show that co-infection with influenza and SARS-CoV-2 leads to more severe outcomes compared to mono-infections, clinical data remains inconclusive, with co-infections exhibiting a wide range of severities (Achdout et al. 2021; Zhang et al. 2021; Maltezou et al. 2023). Thus, we propose that host factors play a crucial role in the heightened susceptibility and severity of co-infection in children and older adults. Age-related differences in immune responses may help explain the varied clinical outcomes of viral co-infections. Although both are vulnerable populations, the immature immune response in children and immunosenescence in older adults can lead to distinct processes of injury repair following infection. With immature adaptive immune memory, children are susceptible to viral co-infection. However, from the perspective of energetic cost theory, children have significant demands for growth and development, and thus they deploy disease tolerance strategies against infections to preserve energy (Harbeson et al. 2018). On the other hand, older adults are prone to severe injury due to aging organs, harmful non-cross-reactive T cell response and inflammaging (Yunis et al. 2023).

The implementation of either antivirals or vaccination aims to address the time-window gap in the primary immune response when patients are initially exposed to the virus. Following vaccination, long-lived memory T cells and B cells with antigen-specificity will develop, leading to a more rapid adaptive response to clear virus during subsequent infections (Mettelman et al. 2022). A retrospective study in the United States during the 2021–2022 flu season revealed that among patients who died from influenza and SARS-CoV-2 co-infection, none had received the influenza vaccination and only one were prescribed with anti-influenza medication (Adams et al. 2022). An in vivo study demonstrated that pre-existing immunity to influenza could prevent mortality from influenza and SARS-CoV-2 co-infection in mice, whereas immunity to SARS-CoV-2 did not confer the same protective effect (Achdout et al. 2021). Hence, multivalent vaccines that combine influenza and SARS-CoV-2 components are deemed necessary for vulnerable populations such as children and the elderly (Wang et al. 2024). Moreover, early administration of antivirals against influenza and SARS-CoV-2 is recommended. In an in vitro model of SARS-CoV-2 and influenza co-infection, monotherapy with either anti-SARS-CoV-2 or anti-influenza medication was found to be less effective in inhibiting the replication of both viruses compared to combined therapy (Liu et al. 2024). However, administration of the anti-influenza drug oseltamivir in a co-infection model of human airway epithelium was able to counteract the interference exerted by influenza on SARS-CoV-2 and promote the replication of SARS-CoV-2 (Cheemarla et al. 2024). The recent report on a severe co-infection human case supported the efficacy and safety of anti-SARS-CoV-2 and anti-influenza combined therapy (Jin et al. 2024).

Due to the differing immunological exposure histories of children and the elderly, laboratory results may only partially reflect actual immune responses in clinical settings. This discrepancy contributes to research gaps regarding virus-host interactions and the interplay between epithelial cells and immune cells at the extremes of age. Further exploration of immunological factors contributing to the heightened susceptibility of children and the elderly, along with the identification of potential therapeutic targets, may enhance the recovery and survival rates in cases of severe influenza and SARS-CoV-2 virus co-infection.

## Data Availability

No datasets were generated or analyzed in this work.
